# Simultaneous Small Bowel Obstruction and Cecal Volvulus From an Internal Hernia Caused by Gastric Band Tubing: A Case Report

**DOI:** 10.7759/cureus.103479

**Published:** 2026-02-12

**Authors:** Seon Woo Kim, Nikhil Patel

**Affiliations:** 1 General Surgery, St. Vincent's Medical Center, Indianapolis, USA; 2 Colorectal Surgery, St. Vincent's Medical Center, Indianapolis, USA

**Keywords:** adjustable gastric band complications, bariatric surgery complications, closed loop obstruction, internal abdominal hernia, lagb, lagb tube, laparoscopic adjustable gastric band

## Abstract

We present a unique case of a 43-year-old woman with a complex bowel obstruction caused by the connecting tubing of a gastric band placed three years prior. The tubing formed a loop with a twist acting as a noose, causing an internal hernia involving the distal ileum. The surrounding adhesions to the transverse colon also caused her mobile cecum to be distended and to be reflected upward. We managed her complex closed-loop bowel obstruction laparoscopically by lysing adhesions and transecting the gastric band tubing. The patient recovered well without sequelae, and the gastric band was later removed by her bariatric surgeon electively. Laparoscopic adjustable gastric band (LAGB) placement used to be one of the most popular forms of bariatric surgery. It is important for abdominal surgeons to be able to recognize various surgical complications of LAGB.

## Introduction

Bariatric surgery remains a durable, popular treatment for morbid obesity, even with the rising popularity of medical therapy such as glucagon-like peptide-1 (GLP-1) agonists. With more than 250,000 bariatric procedures performed each year in the United States [1], not only bariatric surgeons but also any abdominal surgeon frequently encounter surgical patients with a history of bariatric surgery. Although the laparoscopic adjustable gastric band (LAGB) has steadily fallen out of favor, it used to be one of the most popular forms of bariatric surgery performed, accounting for 35.4% in 2011 and 20.2% in 2012 [2]. Either the band itself or the tubing that connects the band to the subcutaneous port can result in surgical complications, even years postoperatively. Thus, it is important for abdominal surgeons to be well-acquainted with potential complications of bariatric surgeries, including the once-popular LAGB. We present a late complication of LAGB: simultaneous small bowel obstruction and cecal volvulus from an internal hernia caused by the gastric band connecting tubing.

## Case presentation

**Figure 1:**
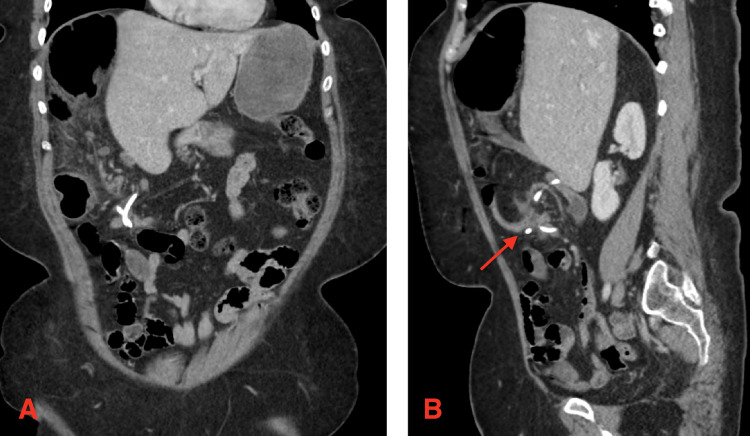
Cross-sectional slides representative of the complex internal hernia. Panel A: Gastric band tubing with a twist. Panel B: Cecum is seen in the right upper quadrant. Arrow points to the mesenteric swirling associated with the distal ileum, transverse colon, and the gastric band tubing. There is no evidence of free intraperitoneal air or pneumatosis intestinalis.

A 43-year-old woman with a history of LAGB placement at a different institution three years prior presented with four days of mid-lower abdominal pain. She did not have chills, nausea, or episodes of emesis. The last bowel movement had occurred one day prior, and she was still passing flatus. On exam, she was afebrile and hemodynamically stable. Abdominal exam was soft with mild tenderness in the lower quadrants without evidence of peritonitis. Laboratory studies showed no leukocytosis or other significant abnormalities. Representative slides of CT abdomen and pelvis with IV contrast are shown here in Figure 1.

Due to the radiographic findings of cecal volvulus, the colorectal surgery service was consulted. No in-house bariatric surgeon was available, but we did discuss surgical options with the bariatric team. Given the clear surgical nature of her problem and potential bowel compromise, we decided to promptly proceed with surgery after obtaining informed consent. Since the initial bariatric surgery was performed at a different institution, we discussed the possible need for additional procedures by her bariatric team to definitively manage her gastric band moving forward.

**Figure 2:**
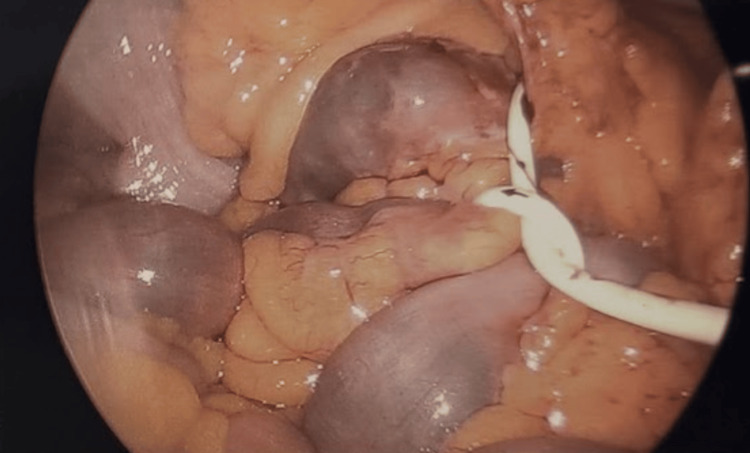
Internal hernia associated with a twisted loop formed by the gastric band tubing.

After establishing pneumoperitoneum and placing 5 mm laparoscopic trocars in the left hemi-abdomen, we followed the gastric band tubing rising from the left upper quadrant down to the right abdomen. We found that the silicone tubing had formed a twisted loop associated with the small bowel, forming an internal hernia. Representative intraoperative image is shown in Figure 2.

**Figure 3:**
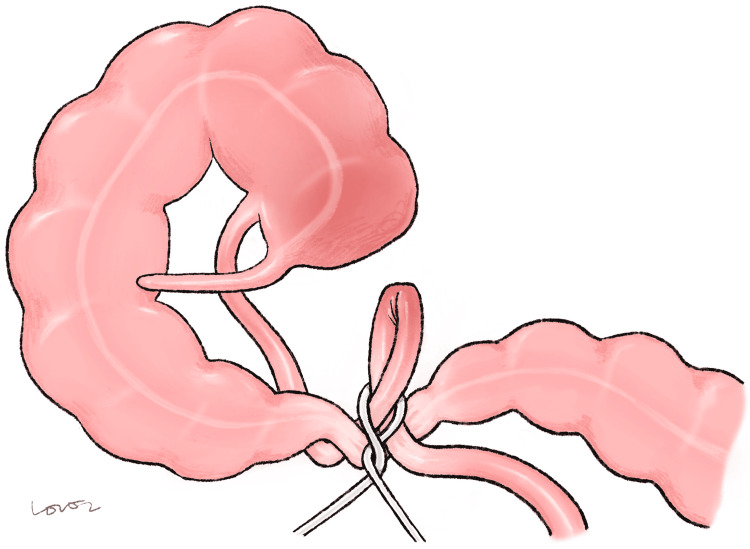
Illustration of the internal hernia based on radiographic and intraoperative findings. Image Credits: Original creation by Hayeon Lee. The image is dedicated for academic purposes only. Permission was obtained from the original creator for reproduction.

The cecum was very mobile and had reflected up into the right upper quadrant. We later discovered that the transverse colon was narrowed due to adhesions, causing her mobile cecum to be distended and then twist up into the right upper quadrant. All visible bowel at this point appeared viable. Representative illustration of the complex bowel obstruction is shown in Figure 3.

After reflecting the cecum and terminal ileum back into the right lower quadrant, we carefully attempted to reduce the internal hernia by sharply lysing the adhesions associated with the small bowel. We also attempted to loosen the strangulating noose formed by the twist in the gastric band tubing. As these maneuvers were unsuccessful, we eventually transected the tubing close to the twist. We immediately saw the tension on the tubing release. We were then able to run the small bowel and confirm its viability and integrity of the mesentery. The adhesion involving the transverse colon was also sharply released. All of the small and large bowel was viable at the end, and no bowel resection was performed. The procedure was completed laparoscopically with minimal blood loss.

The patient recovered expeditiously. Her abdominal pain resolved. Once she tolerated a regular diet and demonstrated resumption of bowel function, she was discharged on postoperative day 1. She subsequently followed up with her bariatric surgeon. Two months following this hospitalization, her gastric band and port were removed. She was already contemplating removal of her LAGB prior to her acute presentation as she had achieved her weight loss goal and behavioral modifications. She has since recovered well without sequelae.

## Discussion

The complication rate of LAGB requiring removal or reoperation is reported to be approximately 40% [3,4], with reports of an even higher rate [5]. Inadequate weight loss is the most commonly reported reason for band removal [6], among others, such as intolerance, band erosion, and slippage [7]. Although there are reports of complications caused by the gastric band connecting tubing, this remains rare [8]. It is even more rare to have an internal hernia associated with both small bowel obstruction and cecal volvulus. With this case report, we aim to highlight the importance of early recognition and intervention for a complex surgical complication caused by a now less-popular bariatric surgery.

Early recognition can be achieved by thorough history-taking and an increased index of suspicion. Patients with LAGB do not always present with emesis since the band can prevent regurgitation [8]. Cross-sectional imaging can be instrumental in diagnosis, as in our case. Even though the colorectal surgery service in our institution was consulted for the diagnosis of cecal volvulus, the patient’s mobile cecum was likely reflected up into the right upper quadrant as a result of the complex adhesions involving the gastric band tubing. We believe that the twist in the tubing resulted in an internal hernia of the distal small bowel, subsequently forming adhesions to the adjacent transverse colon. The colon proximal to this became distended, and eventually the patient's mobile cecum was reflected upwards, causing it to appear as a volvulus. To our knowledge, there are no case reports that describe an internal hernia from the gastric band tubing that concurrently involves both the small and large intestine.

With early intervention, all bowel was salvaged, and we were able to complete the procedure laparoscopically with 5 mm laparoscopic ports. A recent literature review revealed that laparotomy was necessary in 41% of cases to manage bowel obstruction resulting from gastric banding - laparotomy rate was higher with tubing-related complications than with the band [8]. Here, we emphasize the importance of early intervention to avoid bowel necrosis and more extensive intervention.

We elected to leave the gastric band in place because we felt that the patient would be best served with further management of her device under the care of a bariatric surgeon, either with removal or salvage of the gastric band. Multiple ways of salvaging the gastric band after transecting the tubing have been described in the literature. The tubing can be connected back to the port or even be re-routed to a new location [9-11]. Our patient had excellent excess weight loss with her LAGB and was already contemplating its removal even prior to her acute presentation to our service.

## Conclusions

LAGB placement is a less popular surgical option for treating morbid obesity, but surgeons still encounter complex complications from it. With this case report, we aim to contribute to the body of literature that describes various surgical complications of LAGB. It is important for abdominal surgeons to be able to promptly recognize and treat such complications of LAGB.

## References

[1] (2026). Estimate of Bariatric Surgery Numbers, 2011-2023. https://asmbs.org/resources/estimate-of-bariatric-surgery-numbers/.

[2] (2026). Metabolic and Bariatric Surgery. https://asmbs.org/resources/metabolic-and-bariatric-surgery/.

[3] Lazzati A, De Antonio M, Paolino L, Martini F, Azoulay D, Iannelli A, Katsahian S (2017). Natural history of adjustable gastric banding: Lifespan and revisional rate: A nationwide study on administrative data on 53,000 patients. Ann Surg.

[4] Shen X, Zhang X, Bi J, Yin K (2015). Long-term complications requiring reoperations after laparoscopic adjustable gastric banding: A systematic review. Surg Obes Relat Dis.

[5] Toolabi K, Golzarand M, Farid R (2016). Laparoscopic adjustable gastric banding: Efficacy and consequences over a 13-year period. Am J Surg.

[6] Jensen MD, Ryan DH, Apovian CM (2014). 2013 AHA/ACC/TOS guideline for the management of overweight and obesity in adults: A report of the American College of Cardiology/American Heart Association Task Force on Practice Guidelines and The Obesity Society. J Am Coll Cardiol.

[7] Cho EJ, Kim SM (2019). Explantation of adjustable gastric bands: An observation study of 10 years of experience at a tertiary center. Yonsei Med J.

[8] Vitiello A, Matarese A, Sansone G (2024). Reports of gastric banding and bowel obstruction: A narrative review of the literature. J Clin Med.

[9] Sharma K, Arfan S, Thota SS, Agbasi C, Khan L, Naqvi L, Tiesenga F (2023). Small bowel obstruction secondary to laparoscopic adjustable gastric band connecting tube intertwinement within the mesentery: A case report. Cureus.

[10] Oppliger F, Wiedmaier G, León J (2014). Acute small bowel obstruction due to the connecting tube of a gastric band. Surg Obes Relat Dis.

[11] DeNino WF, Forgione PM (2010). Small bowel obstruction from small bowel volvulus and gram-positive peritonitis in laparoscopic adjustable gastric banding. Surg Obes Relat Dis.

